# Hypoglycemia caused by co-secretion of insulin from lung tumor and cardia cancer: first case report

**DOI:** 10.1590/1516-3180.2017.0136060617

**Published:** 2017-11-17

**Authors:** Yaning Chen, Yi Kang, Liu Hong, Hebin Yao

**Affiliations:** I Attending Physician, Department of Endocrinology, General Navy Hospital of PLA, Beijing, China.; II MD. Professor, Department of Endocrinology, General Navy Hospital of PLA, Beijing, China.; III MD. Professor, Department of Pathology, General Navy Hospital of PLA, Beijing, China.

**Keywords:** Hyperinsulinism, Adenocarcinoma of lung, Diazoxide

## Abstract

**CONTEXT:**

Non-islet-cell-tumor-induced hypoglycemia (NICTH) is caused on rare occasions by secretion of insulin from tumor cells that are reported to have a single tissue origin.

**CASE REPORT:**

A 67-year-old male patient had cardia adenocarcinoma and concomitant lung adenocarcinoma with extensive metastases and repeated episodes of intractable hypoglycemia. Immunohistochemical staining for insulin showed that lung adenocarcinoma stained positive and gastric cardia adenocarcinoma stained weakly positive. These results indicate that tumor cells of different tissue origins co-secreted insulin.

**CONCLUSIONS:**

This is the first report on intractable hypoglycemia due to co-secretion of insulin from two kinds of primary tumor cells in a single patient.

## INTRODUCTION

Non-islet-cell-tumor-induced hypoglycemia (NICTH) is a rare paraneoplastic syndrome characterized by repeated episodes of hypoglycemia. NICTH is commonly associated with excessive secretion of immature insulin-like growth factor (IGF)-2 precursor or IGF-1, by mesenchymal or epithelial tumor cells. Several studies have also reported that NICTH is related to excessive secretion of insulin from some tumors originating from a single tissue. Here, we report the first case of intractable hypoglycemia due to co-secretion of insulin from gastric cardia adenocarcinoma (GCA) and lung adenocarcinoma (LA), as confirmed by immunohistochemical staining for insulin. We obtained approval from our institution’s ethics committee to report this case and the patient’s family consented to the publication.

## CASE REPORT

A cardia mass was found in a 67-year-old male patient in May 2012, and surgical resection was performed. Postoperative pathological examination showed moderately to poorly differentiated ulcerative gastroesophageal junction (GEJ) adenocarcinoma. In March 2015, the patient complained of frequent dizziness in the mornings, which improved after eating. On the morning of May 4, 2015, he presented limb convulsion unconsciously and could not be awakened. The patient’s blood glucose level was 0.9 mmol/L. Five minutes after 50% glucose treatment, he recovered consciousness. Positron emission tomography-computed tomography (PET-CT) showed pulmonary, adrenal, intracranial, intrahepatic, retroperitoneal and thoracic vertebral lesions.

**Figure 1:**
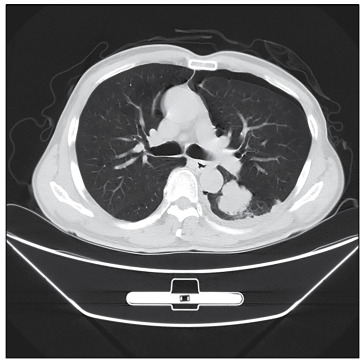
Computed tomography scan showing a mass in lung window.

On May 11, the patient underwent CT-guided biopsy of a lesion in the left lung (Figure 1). Pathological examination showed lung adenocarcinoma (LA), and he received stereotactic radiotherapy in the whole brain, lungs and abdominal cavity. He and his family refused chemotherapy and further surgery. During the treatment, hypoglycemia occurred many times. At first, extra meals could maintain normal blood glucose. Later, continuous intravenous infusion of glucose injection was required, while the glucose concentration and infusion rate progressively increased. To prevent hypoglycemia, the maximum infusion rate for 50% glucose injection was 100 ml per hour. Dynamic enhanced MRI did not show any clear lesion in the pancreas. Adrenocorticotropic hormone, cortisol, growth hormone, glucagon and five thyroid function parameters were within normal ranges. An insulin autoantibody test was negative. IGF-1 was 222 (96-212 ng/ml) and IGF-2 was significantly lower than normal according to western blotting. The patient was unresponsive to diazoxide (125 mg, three times a day for 10 days), and at that time, his blood diazoxide concentration was 13.4 µg/ml. The treatment was subsequently changed to tacrolimus capsules (10 mg, once a day), but the patient died five days later. The patient’s family refused to allow an autopsy.

**Figure 2:**
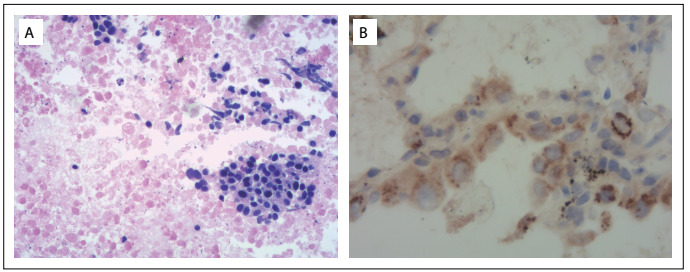
Immunohistochemical staining on lung adenocarcinoma in our patient: (A) hematoxylin-eosin staining on lung adenocarcinoma (× 200); and (B) tumor cells of lung adenocarcinoma with positive staining for insulin (× 200).

**Figure 3:**
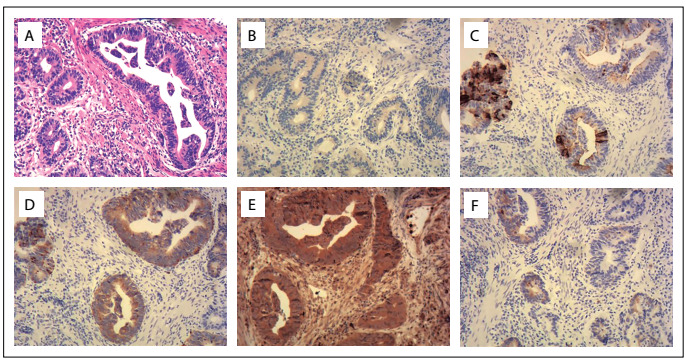
Immunohistochemical staining on gastroesophageal junction adenocarcinoma in our patient: (A) hematoxylin-eosin staining on gastroesophageal junction tumor cells (× 100); (B) tumor cells stained weakly positive for insulin (× 100); and (C-F) tumor cells stained positive for CgA, syn, GPG9.5 and MAP2abc (× 100).

Immunohistochemical staining for insulin showed that LA samples were stained positive (Figure 2B) and GCA samples weakly positive (Figure 3B). The results from immunohistochemical staining on GEJ adenocarcinoma were as follows: CgA (+), syn (+), GPG9.5 (+) and MAP2abc (+) (Figure 3 C-F).

## DISCUSSION

In NICTH, there are mainly three mechanisms leading to hypoglycemia: tumor cells secrete excessive high-molecular-weight IGF-2 precursor, IGF-1, and insulin.^1^ Previous studies have reported that insulin-secreting non-islet-cell tumors can originate in any germ layer, and that all of them have a single tissue origin. In the present study, the patient had two kinds of tumors that originated from the endoderm, i.e. GCA and LA, and both of them secreted insulin.

**Figure 4:**
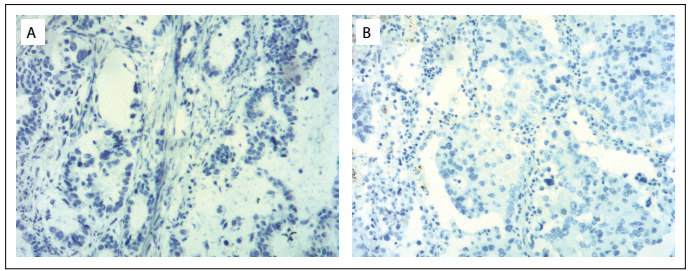
Immunohistochemical staining on gastroesophageal junction adenocarcinoma and lung adenocarcinoma in the control: (A) tumor cells of gastroesophageal junction adenocarcinoma with negative staining for insulin (× 100); and (B) tumor cells of lung adenocarcinoma with negative staining for insulin (× 100).

When hypoglycemia occurred in our patient, both serum C-peptide and insulin levels increased. The patient had hypoglycemia due to endogenous hyperinsulinism. Drug-induced hypoglycemia and insulin autoimmune syndrome were ruled out. Our patient had GCA and concomitant LA, thus suggesting the possibility of ectopic secretion of insulin from non-islet-cell tumors. Immunohistochemical staining for insulin showed that LA stained positive and GCA stained weakly positive, thus indicating that high levels of endogenous insulin were secreted from both GCA and LA. The control tumor tissue was stained negative for insulin (Figure 4). Additionally, GCA was stained positive for neuroendocrine cell-specific markers (syn and CgA), which showed that GCA belongs to neuroendocrine cells and further supports the notion that GCA secreted insulin. Thus far, ectopic secretion of insulin has been reported in only a few non-islet-cell tumors, and all of these had a single tissue origin in different germ layers. Immunohistochemical staining on a pulmonary lesion showed CKAE1/AE3 (+++), CK7 (+++), TTF-1 (+++), napsin-A (++) and Ki67 labeling index of 10%. TTF-1 and napsin-A-positive results showed that the pulmonary lesion was not metastatic GCA, but was a primary LA. Thus, our patient was diagnosed as having tumors from two kinds of tissue cells of endodermal origin. Hence, our patient’s high insulin levels came from tumors of two different tissue origins. This is the first reported case of NICTH caused by co-secretion of insulin from multiple primary carcinomas.

Diazoxide can inhibit secretion of insulin and ease the condition of hypoglycemia. In 1979, Fajans used diazoxide for the first time to treat insulinoma.^2^ Diazoxide was also later used to treat hypoglycemia caused by high levels of insulin secreted from inoperable extrapancreatic malignancies. Gill et al.^3^ found that the mean dose of diazoxide for treating islet cell tumors was 267 ± 138 mg/day (range 100-600), with a response rate of 97.5%. Our patient had absolute indications for taking oral diazoxide tablets (125 mg, three times a day). However, this did not relieve his hypoglycemia. His plasma concentration of diazoxide was 13.4 µg/ml, which showed that diazoxide did not work. Shames et al.^4^ reported on a patient with bronchial carcinoid tumors who had severe hypoglycemia relating to hyperinsulinism. The efficacy of treatment with diazoxide injection was poor. The authors speculated that this was associated with unusual autonomy of insulin secretion by tumor cells. In addition to this, we speculate that the molecular mechanism through which non-islet-cell tumors secrete insulin probably differs from that of islet cell tumors. Our patient’s treatment was then changed to tacrolimus capsules. Tacrolimus has been reported to inhibit hyperglycemia-stimulating insulin gene expression with an inhibition rate of up to 70%. However, our patient’s condition deteriorated sharply and he died after five days of treatment with tacrolimus.

We reviewed the literature in MEDLINE and EMBASE using the English keywords “Hyperinsulinism”, “Hypoglycemia”, “Cardia neoplasms” and “Lung neoplasms” (Table 1). No other similar case was found.

**Table 1:** Search of the literature in medical databases for case reports on insulin secretion from lung carcinoma and cardia carcinoma. The search was conducted on June 10, 2017

Database	Search strategy	Full result	Similar case report
MEDLINE (via PubMed, June 10, 2017)	#1 (“Cardia Neoplasms”[Mesh]) OR “Lung Neoplasms”[Mesh])) #2 “Hyperinsulinism”[Mesh]) #3 “Hypoglycemia”[Mesh] #4 #1 AND #2 AND #3	3	0
Embase (via Embase, June 10, 2017)	#1 (‘Cardia carcinoma’/exp/mj) OR (‘Lung carcinoma’/exp) #2 ‘Hyperinsulinism’/exp/mj #3 ‘Hypoglycemia’/exp/mj #4 #1 AND #2 AND #3	0	0

## CONCLUSION

In summary, this is the first reported case of hypoglycemia associated with co-secretion of insulin by LA and GCA. Regardless of the type of tumor tissues, NICTH should be taken into consideration for some nonspecific symptoms of hypoglycemia in tumor patients, such as dizziness, convulsions, hallucinations and coma. Early diagnosis and timely treatment are recommended for these patients, to improve their quality of life.
